# Dissection of the Genetic Association between Anorexia Nervosa and Obsessive–Compulsive Disorder at the Network and Cellular Levels

**DOI:** 10.3390/genes12040491

**Published:** 2021-03-27

**Authors:** Weichen Song, Weidi Wang, Shunying Yu, Guan Ning Lin

**Affiliations:** School of Biomedical Engineering, Shanghai Jiao Tong University, Shanghai 200030, China; goubegou@sjtu.edu.cn (W.S.); wwd-swxx@foxmail.com (W.W.); yushuny@yahoo.com (S.Y.)

**Keywords:** anorexia nervosa, co-morbidity, network analysis, obsessive-compulsory disorder, risk genes

## Abstract

Anorexia nervosa (AN) and obsessive–compulsive disorder (OCD) exhibit a high co-morbidity rate, similar symptoms, and a shared genetic basis. However, an understanding of the specific underlying mechanisms of these commonalities is currently limited. Here, we collected Genome-Wide Association Analysis results for AN and OCD, and obtained genes hit by the top SNPs as the risk genes. We then carried out an integrative coexpression network analysis to explore the convergence and divergence of AN and OCD risk genes. At first, we observed that the AN risk genes were enriched in coexpression modules that involved extracellular matrix functions and highly are expressed in the postnatal brain, limbic system, and non-neuronal cell types, while the OCD risk genes were enriched in modules of synapse function, the prenatal brain, cortex layers, and neurons. Next, by comparing the expressions from the eating disorder and OCD postmortem patient brain tissues, we observed both disorders have similar prefrontal cortex expression alterations influencing the synapse transmission, suggesting that the two diseases could have similar functional pathways. We found that the AN and OCD risk genes had distinct functional and spatiotemporal enrichment patterns but carried similar expression alterations as a disease mechanism, which may be one of the key reasons they had similar but not identical clinical phenotypes.

## 1. Introduction

Anorexia nervosa (AN) and obsessive–compulsive disorder (OCD) are two distinct neuropsychiatric disorders but share a genetic basis [1,2,3]. Epidemiological studies have found that the diagnosis of OCD would increase the risk of AN in later life (risk ratio = 3.6) and vice versa (risk ratio = 9.6) [4]. Similarly, the prevalence of OCD is higher in AN patients than in the population [5]. The prevalence of AN is also higher in OCD patients [6], which indicates significant co-morbidity between the two diseases. In accordance with the epidemiological associations, clinical studies revealed that AN and OCD have highly similar phenotypes, such as perfectionism, rigidity, persistency, obsessive thoughts [7], excessive [8], and ritual behaviors [9]. Clinicians have also illustrated the characteristic phenotypes shared by AN and OCD: temporary relief for anxiety after obsessive–compulsive behaviors, followed by increasing long-term anxiety and fear [10]. These significant overlaps between AN and OCD has led some researchers to view them as part of an integrated spectrum, obsessive–compulsive spectrum disorders [11], indicating that they may be the different phenotypes of an identical pathology.

Genetic studies on AN and OCD have also suggested that they have shared underlying mechanisms. A case–control study has found a significant familial aggregation of AN and OCD [12], and the bivariate twin models from the Swedish National Patient Register have reported a significant genetic overlap between the two diseases [4], with a correlation coefficient of 0.52. Consistently, Linkage Disequilibrium Regression analysis using Genome-Wide Association Study (GWAS) data revealed a positive genetic correlation [13,14]. The most recent meta-analysis from Psychiatric Genomics Consortium [15] has revealed that AN and OCD shared a compulsive component of genetic architecture, which contributed to their significant genetic correlation (the correlation coefficient reached 0.50) and commonalities in obsessive–compulsive symptoms. These results have suggested that AN and OCD share a large proportion of their genetic basis and indicated that they might have a common pathology.

However, AN and OCD are clinically recognized as different disorders due to their profound difference in multiple aspects. By comparing AN and OCD patients, researchers have found a significant divergence with regard to cognition [16,17], emotion [18,19], and personality [20,21]. Furthermore, choices of treatments, such as deep brain stimulation [22], selective serotonin reuptake inhibitor [23], and cognition-behavior therapy [24], are different between AN and OCD patients. The situation is even complicated and worse in co-morbidity cases [4,24]. One possible explanation is that AN and OCD exhibit distinct genetic and transcriptomic characteristics at the regional and cellular levels, which served as the foundation of the differential response to cognitive intervention and regional stimulation. These discrepancies urge researchers to substantially investigate the convergence and divergence of the AN and OCD pathologies, to better understand them and thus gain more thorough insight into their genetic etiology.

To achieve this task, we have conducted a systematic analysis combining genetic data [25,26] and transcriptomic data [27,28] to dissect the shared and unique components of the genetic pathology of the two diseases. We first collected the risk genes implicated by the SNPs for the two diseases and explored the spatiotemporal, cell-type specific, and functional patterns of these genes at the network as well as genome-wide levels. As a result, we have found distinct and semblable coexpression patterns across developmental periods, cell types, and phenotype conditions in both disorders. So, these analyses provided insights into the convergence and divergence of AN and OCD, which could help further functional analysis and clinical personal medicine.

## 2. Materials and Methods

### 2.1. Data Collection

The brain transcriptome data of the OCD, eating disorder, and control groups were obtained from GSE60190 [28]. This dataset, provided by Jaffe et al., includes normalized expression array data of the prefrontal cortex from patients with eating disorders (ED; including three AN, seven bulimia nervosa patients, two binge–purge type of AN, and three eating disorder not otherwise specified), obsessive–compulsive disorder/obsessive–compulsive personality disorder or tics (OCD/OCPD/Tic; *N* = 16), and non-psychiatric controls (*N* = 102). We did not perform extra data preprocessing but reran the differential expression analysis (see section “Differential Expression Analysis”). 

The GWAS summary data for AN was collected from Watson et al. [25]. We considered all Linkage Disequilibrium (LD) blocks with a genome-wide significant (*p* < 5 × 10^−8^) tag SNP as the target blocks and extracted all genes within the target blocks as the risk genes of AN. The range of LD blocks was estimated by the original GWAS by local LD structure and a *p*-value threshold. Similarly, the LD blocks for OCD were downloaded from Arnold et al. [26] (Table 1 of ref [26]; tagged region by the index SNP at r^2^ > 0.2 with 1KG as the reference panel) and the OCD risk genes were retrieved from the blocks, respectively. Only SNPs passing quality control in at least 500 cases and 500 controls were retained. Since this GWAS had limited statistical power, we set the significance threshold at 1 × 10^−5^ for the target blocks. To minimize the impact of false-positive risk genes, we excluded the most marginal genes for the large blocks. A total of 92 AN risk genes and 51 OCD risk genes were obtained. We downloaded brain transcriptome data during normal brain development from the BrainSpan portal [27].

### 2.2. Weighted Gene Co-Expression Network Analysis (WGCNA)

We divided the BrainSpan v1.0 [27] RPKM data, which spans 15 consecutive periods of neurodevelopment and adulthood from 5.7 postconceptual weeks (PCW) to 82 years. We used two subsets of prenatal and postnatal samples. For both subsets, only genes with non-zero RPKM in at least half of the samples and a coefficient of variance >0.3 were retained for WGCNA and were log10-transformed. A soft threshold was set at 12 for the prenatal subset and 16 for the postnatal subset by the pickSoftThreshold function in the WGCNA v1.70 [29] R package. Next, we built up the signed networks by the blockwiseModules function in WGCNA R package and partitioned genes into modules with a tree cut height of 0.3, minimum module size = 20, and maximum module size = 1000. We calculated the first principal component of each module, namely Module Eigengene (ME), to represent the overall expression levels of the module in each sample. To compare the temporal trajectory of each module, we fit a curve for ME across different developmental periods using the loess function in R v4.0.0.

After the detection of coexpression modules for both subsets, we tested if the AN and OCD risk genes were enriched in any of the modules by Fisher’s exact test. We removed the risk genes that were not presented in the BrainSpan data. The modules with the lowest *p*-value were extracted for further analysis. Since the OCD risk genes showed enrichments in one prenatal module (M6) and one postnatal module (M17), we performed EWCE v1.0 (see Section 2.4. “spatiotemporal expression analysis”) on the intersection of these two modules and found that they were more highly expressed in the prenatal period. Thus, we chose prenatal M6 for further analysis. Similarly, for the AN risk genes, we chose postnatal M2 for the following analysis. As a negative control, we repeated the enrichment analysis by genes related to height and BMI curated at traitDB (https://atlas.ctglab.nl/traitDB) (accessed on 10 March 2021). A lack of significant enrichment after *p*-value adjustment (nominal *p* > 0.05) was considered the evidence that our enrichment analysis did not introduce false-positive results.

### 2.3. Gene Ontology (GO) Analysis

We performed a GO enrichment analysis of the gene lists we were interested in by ClusterProfiler v3.12 [30] in the R package. We tested the genes of interest enriched in any GO-BP pathway by the hypergeometric test. Gene background was defined as all genes with a GO annotation. Only pathways with more than ten genes were included in our analysis. The *p*-values of the hypergeometric tests were adjusted for multiple testing by the Benjamin–Hochberg method. We only retained the pathways with an adjusted *p* < 0.05, size > 20 and < 500.

### 2.4. Spatiotemporal Expression Analysis

To analyze the expression patterns of the AN and OCD risk genes across different developmental periods and brain regions, we applied the EWCE v1.0 R package [31] on BrainSpan data, which is an enrichment tool that performed the bootstrap test on the gene specificity matrix based on BrainSpan data. The developmental periods were defined as previously described [32]: early embryo (8–12 weeks), the mid-early embryo (13–16 weeks), the mid-late embryo (17–21 weeks), and late embryo (24–37 weeks) for prenatal data; and infancy (born 3 years), childhood (4–11 years), adolescence (13–19 years), and adult (19 years) for postnatal data. Specificity was defined in the corresponding paper [31]. We first calculated the specificity matrix of the BrainSpan datasets by the “generate.celltype.data” function. Then, the enrichment of genes within the prenatal module M6 and postnatal module M2 was tested by the “bootstrap.enrichment.test” function with the number of bootstraps = 10,000. We then defined the background gene list as all genes annotated in the BrainSpan dataset.

### 2.5. Network Analysis

To construct the coexpression network for the prenatal module M6 and postnatal module M2, we first calculated the signed Topology Overlap Measures (TOM) [29] for both modules. Then, we retrieved all the genes that connected to at least one risk gene with TOM ≥ 0.1. These genes, together with the risk genes of AN or OCD, were used to construct the network with all connections between them (only connections with TOM ≥ 0.1). Hub genes were identified by two measurements: degree score within the network, and the module eigengene connectivity (kME, defined as the correlation coefficient between gene expression value and the module eigengene). The GO annotations were retrieved from the clusterProfiler v3.12 [30] R package. 

### 2.6. Differential Expression Analysis

We obtained postmortem cortex transcriptome data from Jaffe et al. [28] (GSE60190) and compared the gene expression of the ED and OCD patients to that of a healthy control. GSE60190 contains normalized Illumina HumanHT-12 v3 (San Diego, CA, USA) microarray data collected on postmortem dorsolateral prefrontal cortexes. The acquisition and process of data from the case and control were performed similarly. We applied limma [33] on this transcriptome data to identify the genes that were significantly dysregulated in the cortex of patients with ED or OCD. We first fit a linear model with the following design, which controlled all the provided covariates and compared the ED and OCD groups to the control:

Expression ~ intercept + group + RIN + Ph + age + PMI + sex

Then, we computed empirical Bayesian statistics for comparison between the ED and control groups as well as between the OCD and control groups. We extracted all the genes with a Benjamini–Hochberg adjusted *p*-value < 0.05 as the Differentially Expressed Genes (DEGs). 

### 2.7. Gene Set Enrichment Analysis

We applied the Gene Set Enrichment Analysis (GSEA) of the Reactome pathways [34] using the ReactomePA v1.34 [35] R package. The input to ReactomePA was the gene list ordered by fold change from the differential expression analysis. GSEA was repeated for both OCD vs control and ED vs control differential expression analysis. We extracted Normalized Enrichment Scores (NES) for each pathway to compare the biological significance between the expression patterns of OCD and ED. GSEA was run with the number of permutations = 10,000. We retained the pathways with an adjusted *p* < 0.05, and size > 20 and < 500.

Using the same ranked gene lists for OCD and ED, we analyzed the expression alteration of the previously identified M6 module (BrainSpan prenatal) and M2 module (BrainSpan postnatal) using the GSEA function from the clusterProfiler v3.12 [30] R package.

## 3. Results

**Figure 1:**
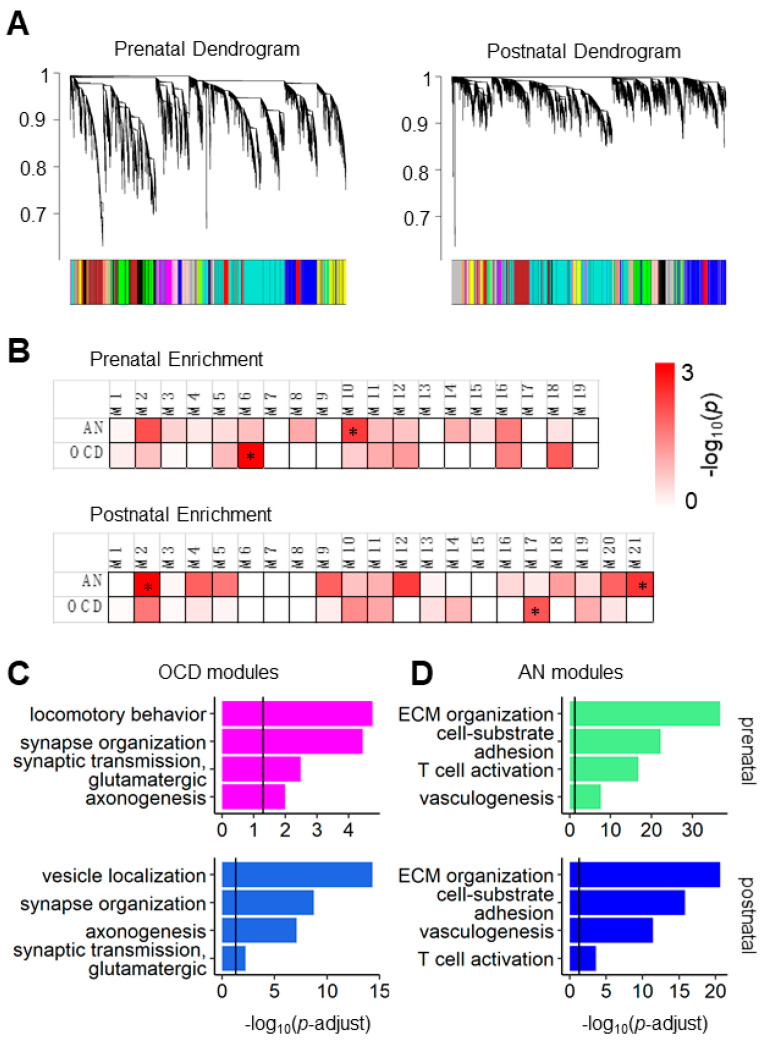
Coexpression and functional enrichments of anorexia nervosa (AN) and obsessive–compulsive disorder (OCD) genes. (**A**) WGCNA dendrogram for the prenatal brain (left) and postnatal brain (right) expression profiles from BrainSpan. (**B**): Enrichment of AN and OCD genes in the prenatal (left) and postnatal (right) coexpression modules. Asterisks denoted modules with the lowest *p* value for each disorder. (**C**,**D**) GO-BP enrichment results for the enriched modules. ECM: extracellular matrix organization.

To investigate the genetic association between AN and OCD, we first collected the implicated risk genes of AN and OCD identified from GWAS summary statistics [25,26] (Table S1). Since AN and OCD shared a profound genetic basis [1,2,3], our first question is whether their risk genes had a similar coexpression pattern. We utilized the spatially and temporally rich transcriptome data set from BrainSpan [27] and constructed coexpression networks for both the prenatal and postnatal periods. As a result, we identified 19 coexpression modules in the prenatal epoch and 21 in the postnatal epoch (Figure 1A). 

### 3.1. AN and OCD Risk Genes Coexpression Modules Show Different Biological Functions in Prenatal and Postnatal Periods

We observed that the AN and OCD risk genes were enriched in different modules in both subsets. In the prenatal network modules, significantly more AN genes than expected were observed in module 10 (OR = 2.44, Fisher’s exact *p* = 0.0066), whereas significantly more OCD genes than expected were observed in module M6 (OR = 9.33, Fisher’s exact *p* = 0.0014) (Figure 1B). For the postnatal analysis, the AN genes were most significantly enriched in the postnatal network M2 module (OR = 2.65, Fisher’s exact *p* = 0.0013) and the OCD genes in the postnatal network module M17 (OR = 2.52, Fisher’s exact *p* = 0.012) (Figure 1B). As a negative control, genes related to height and BMI were not enriched in these modules (*p* > 0.05). These results suggested that the spatiotemporal coexpression patterns of the AN and OCD risk genes during normal brain development was not identical.

Next, we investigated whether modules enriched with the AN or OCD genes differed in biological functions. As shown in Figure 1C, we observed that both prenatal M6 and postnatal M17 modules co-expressed with OCD genes enriched in regulation of neuron projection development (prenatal M6: *p*-adjust = 1.45 × 10^−4^; postnatal M17: *p*-adjust = 1.85 × 10^−9^) and glutamatergic synaptic transmission (prenatal M6: *p*-adjust = 0.003; postnatal M17: *p*-adjust = 0.006). On the other hand, prenatal M10 and postnatal M2 modules co-expressed with AN genes (Figure 1D) had similar functions in extracellular structure organization (prenatal M10: *p*-adjust = 6.60 × 10^−38^; postnatal M2: *p*-adjust = 2.45 × 10^−21^) and immune-related pathways (prenatal M10: neutrophil activation, *p*-adjust = 4.31 × 10^−20^; postnatal M2: defense response to virus, *p*-adjust = 2.02 × 10^−12^).

Since the prenatal and postnatal modules of the same disease had similar biological significance, we asked whether these modules were similarly critical to the disease, or one of them served as the primary module of the disease. As shown in Figure S1C,D, the overlapped genes between prenatal M6 and postnatal M17 were preferentially expressed in the prenatal period (fold change = 1.21, *p* < 0.0001), whereas the overlapped genes between prenatal M10 and postnatal M2 were preferentially expressed in infancy (fold change = 1.33, *p* < 0.0001) and the adolescence period (fold change = 1.14, *p* < 0.0001). These results suggest that the genes co-expressed with the OCD risk genes are highly expressed in the prenatal period, and that with the AN risk genes are highly expressed in the postnatal period. These results confirmed prenatal M6 as the primary module for OCD, and postnatal M2 as the primary module for AN. Next, we focused our analyses on these two modules.

### 3.2. Coexpression Modules of the AN and OCD Risk Genes Exhibited Distinct Spatiotemporal and Cell-Type-Specific Expression Patterns

**Figure 2:**
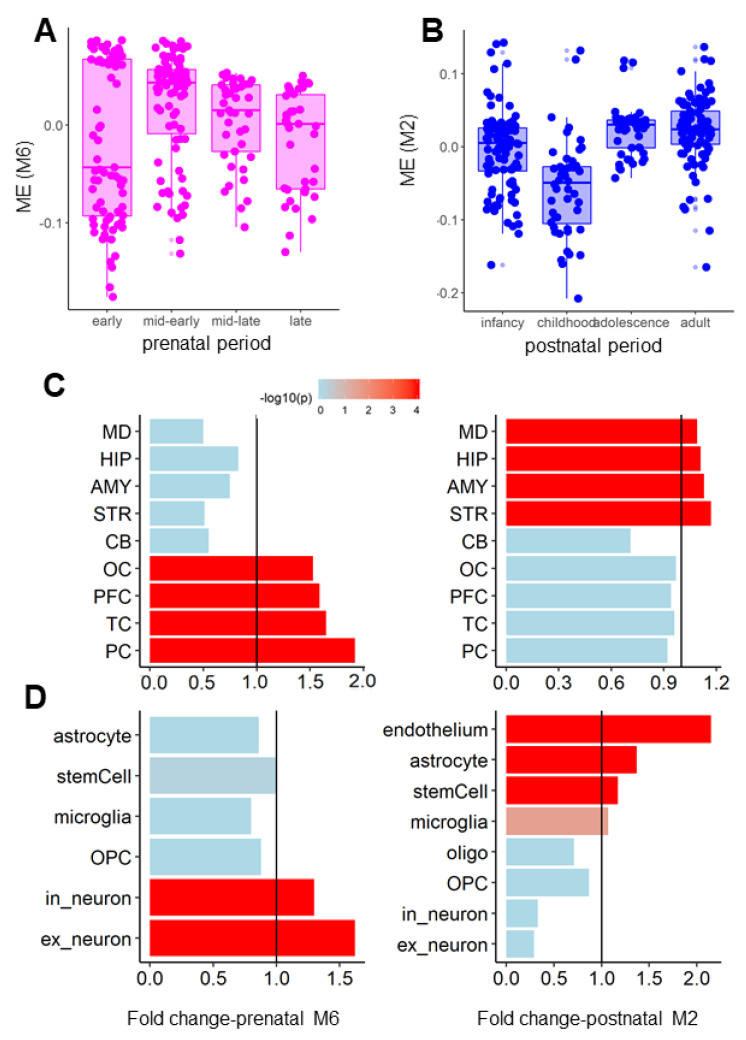
Spatiotemporal and cell-type-specific expression patterns for the enriched modules. (**A**,**B**) Spatial expression patterns for the OCD module (**A**, M6) and AN module (**B**, M2). The y-axis shows the module eigengene. (**C**) Brain region-specific expression patterns for M6 (left) and M2 (right). The x-axis shows the fold change calculated by the EWCE R package; the colors correspond to the *p*-values obtained from the EWCE. (**D**) Cell-type-specific expression patterns for M6 (left) and M2 (right), similar to C. MD: medium dorsal thalamus. HIP: Hippocampus. AMY: Amygdala. STR: Striatum. CB: Cerebellum. OC: Occipital cortex. PFC: Prefrontal cortex. TC: Temporal cortex. PC: Parietal cortex. OPC: oligodendrocyte precursor. Oligo: oligodendrocyte. Ex_neuron: excitatory neuron. In_neuron: inhibitory neuron.

Having identified the primary modules as well as their biological functions for AN and OCD, we next compared these coexpression modules from the aspects of spatiotemporal expression patterns. Using the EWCE analysis technique [31], we found that the genes in prenatal M6 were expressed significantly higher than expected in the mid-early prenatal period (fold change = 1.3, *p* < 0.0001), given the fact that module eigengene (ME) of prenatal M6 peaked at mid-early (Figure 2A). Postnatal M2, however, was highly expressed in infancy (fold change = 1.22, *p* < 0.0001) and adolescence (fold change = 1.06, *p* < 0.0001) (Figure 2B). A divergence between the modules was also observed for brain regions, where prenatal M6 was enriched in the cerebral cortex (*p* < 0.0001 for all frontal, parietal, temporal, and occipital cortexes; Figure 2C) and postnatal M2 was enriched in the limbic system (*p* < 0.0001 for the amygdala, medial thalamus, striatum, and hippocampus; Figure 2D). Lastly, we explored the cell-type-specific expression patterns for these two modules using EWCE. We observed that prenatal M6 was more highly expressed in neurons in the embryo brain than expected (excitatory neuron: fold change = 1.62, *p* < 0.0001; gamma-Aminobutyric acid[GABA]-ergic neuron: fold change = 1.30, *p* < 0.0001; Figure 2D). We also found that postnatal M2 was more enriched in the endothelium than expected (fold change = 2.16, *p* < 0.0001) and in astrocytes (fold change = 1.37, *p* < 0.0001, Figure 2D) of the adult brain. These results suggest that the AN and OCD modules had distinct spatiotemporal and cell-type-specific expression patterns.

### 3.3. OCD and Eating Disorder Led to a Similar Brain Transcriptome Alteration Pattern 

We have found the distinct functional and spatiotemporal characteristics of AN and OCD risk genes using transcriptome data of the healthy human brain. However, these characteristics may not be mirrored by transcriptomic alteration of brains of the patients with diseases [36], since the risk SNPs may influence normal functions by means other than transcriptomic dysregulation [37]. Thus, we further analyzed the cortex transcriptomic alteration of OCD and eating disorder (ED) using data from Jaffe et al. [28].

**Figure 3:**
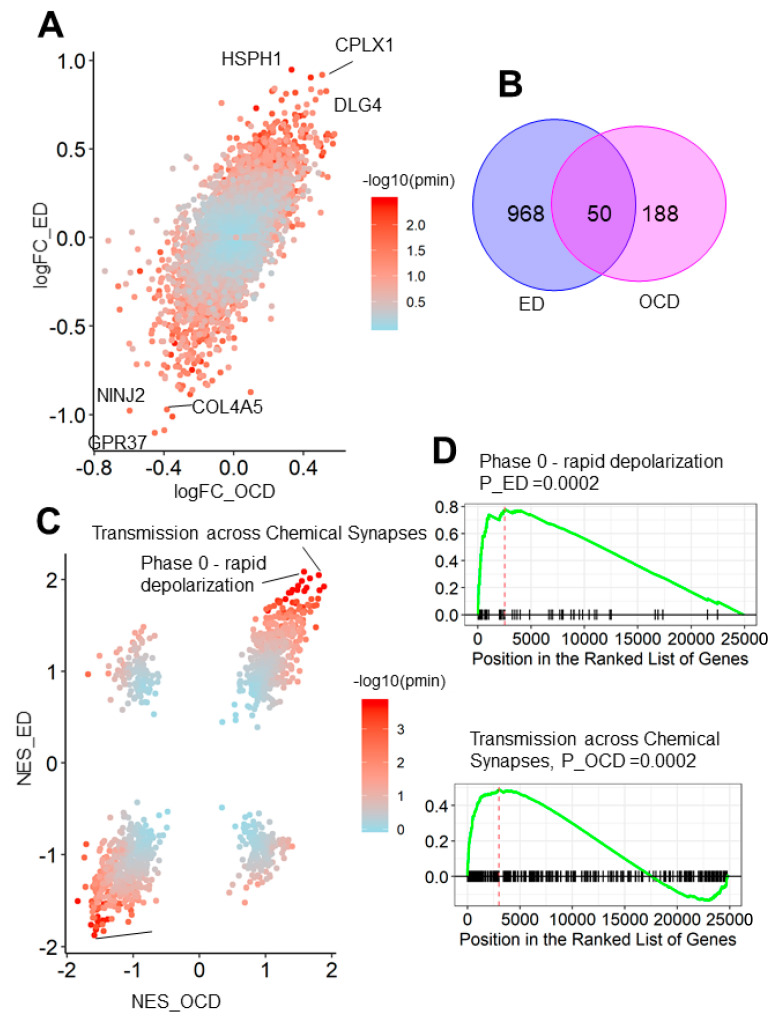
Expression alterations of the prefrontal cortex of ED and OCD patients. (**A**) Differential Expression Analysis for OCD vs. control (X-axis) and ED vs control (Y-axis). Each point corresponded to one gene; colors correspond to the minimal of the *p*-value of OCD vs. control and ED vs. control. (**B**) Venn diagram showing the relation between the differentially expressed genes of ED vs. control (left) and OCD vs. control (right). (**C**) Gene Set Enrichment Analysis (GSEA) results using gene lists ranked by fold change of OCD vs. control (X-axis) and AN vs. control (Y-axis). Each point corresponds to one REACTOME term; colors correspond to the minimal of the *p*-value of OCD vs. control and AN vs. control. (**D**) GSEA plots for top REACTOME terms. NES: Normalized enrichment score. logFC: log fold change. ED: Eating disorder.

Unlike the significant observation in differential coexpression patterns of risk genes in AN and OCD, the log-transformed fold change (logFC) of expression for ED and OCD patients was highly convergent (correlation coefficient = 0.688, *p* < 2.2 × 10^−16^). Among these top differential expression genes, we found upregulated genes involved in synapse organization (*CPLX1*: logFC_OCD = 0.51, logFC_ED = 0.92; *DLG4*: logFC_OCD = 0.53, logFC_ED = 0.83) and downregulated genes involved in cell adhesion (*NINJ2*: logFC_OCD = −0.60, logFC_ED = −0.98; *COL4A5*: logFC_OCD = −0.38, logFC_ED = −0.97). Setting the significance cutoff of *p*-adjust as 0.05, we identified 1118 significant differentially expressed genes (DEGs) for ED and 238 DEGs for OCD. The significant overlapped DEGs count between them (50; p by hypergeometric test <2.2 × 10^−16^, Figure 3B) also supported the fact that transcriptome alteration of ED and OCD were nearly parallel. In summary, ED and OCD brought about similar brain expression alterations.

We further applied GSEA to verify that the functional impacts of the expression alteration were in the same pattern between both diseases (Figure 3C and Table S2). As expected, the Normalized Enrichment Score (NES) was also convergent between two diseases (correlation coefficient = 0.649, *p* < 2.2 × 10^−16^). The upregulated genes of both diseases were enriched in Transmission across Chemical Synapses (NES_OCD = 1.67, NES_ED = 2.01, Figure 3C) and Phase 0—rapid depolarization (NES_OCD = 1.58, NES_ED = 2.08, Figure 3D), supporting the transcriptomic disruption of synapse functions in both diseases. Moreover, for the downregulated genes, the most significant enrichment was found for the SRP-dependent co-translational protein targeting membranes (NES_OCD = −1.59, NES_ED = −1.88, Figure 3D). These findings demonstrated that the convergent functional pathway from the distinct disorders is unmasked by the SNP risk DEGs across ED and OCD patient samples.

### 3.4. LAMB2, HTRA1, and KCTD12 as Hubs for AN and OCD Networks

Having compared the characteristics of AN and OCD genetic etiology from multiple aspects, we next aimed to integrate them into genetic networks so that the whole picture of AN and OCD genetics could be drawn. To achieve this goal, we built up the coexpression network of prenatal M6 for OCD and postnatal M2 for AN by Topology Overlap Measures (TOM, see METHOD).

**Figure 4:**
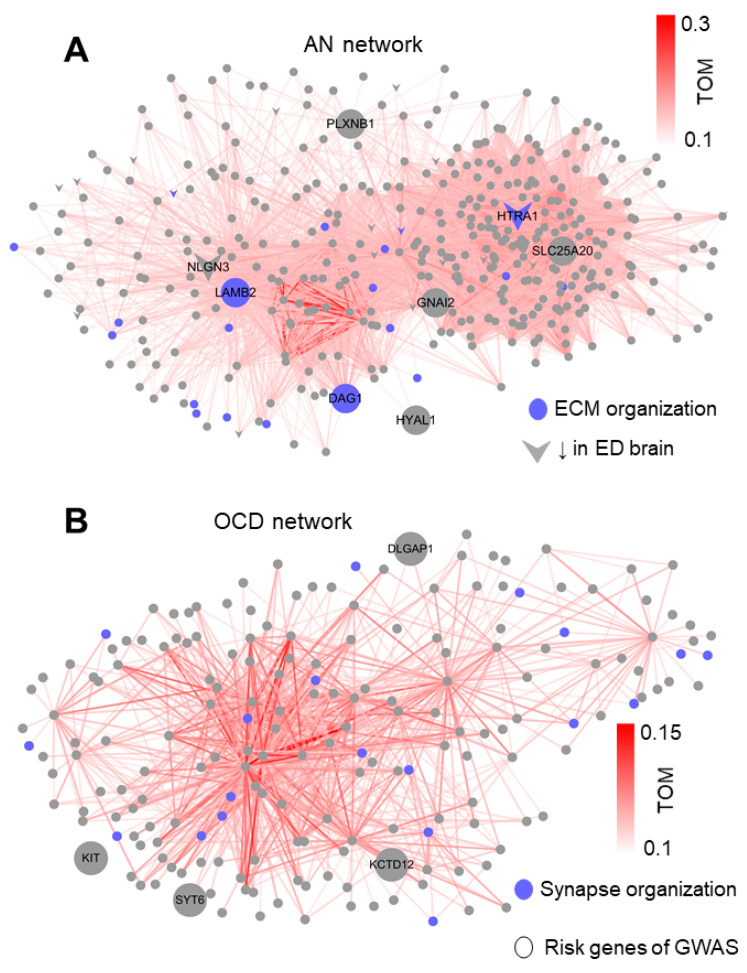
Network analysis for the AN and OCD modules. (**A**) Network for the AN module. (**B**) Network for the OCD module. For each plot, points corresponded to genes within the module, TOM: Topology Overlap Measures, a metric that reflect the coexpression strength between two genes. Only edges with a Topology Overlap Measure (TOM) larger than 0.1 are shown. Risk genes (proxy genes of GWAS top loci) for AN and OCD are shown in large print, with the gene symbols printed. ECM: extracellular matrix organization. ED: Eating disorder.

In the network of AN (postnatal M2, Figure 4A), we found the significant enrichment of AN risk genes (*p* = 0.0013, Figure 1D), genes downregulated in the cortex of ED patients (*p* < 2.2 × 10^−16^), genes involved in extracellular structure organization (*p*-adjust = 2.45 × 10^−21^, Figure 1D), and defense response to virus (*p*-adjust = 2.02 × 10^−12^, Figure 1D). One of the AN risk genes, *LAMB2*, appeared at the center of the network (degree = 154). It also had high intramodular connectivity (kME = 0.82) and was involved in extracellular structure organization, both indicating it as a hub role gene of postnatal M2. Another extracellular structure organization gene, *HTRA1*, had a similar central position (kME = 0.86, degree = 182). Unlike *LAMB2*, *HTRA1* was not impacted by AN risk SNPs but was significantly downregulated in the cortex of ED patients (logFC = −0.89, *p*-adjust = 0.01). As the highest degree of SLC25A20 reached 194, which encodes a mitochondrial solute carrier, it may also be considered a hub risk gene (kME = 0.81).

In the network of OCD (prenatal M6, Figure 4B), we also found the significant enrichment of OCD risk genes (*p* = 0.0014, Figure 1C) and synapse organization (*p*-adjust = 3.55 × 10^−5^), but not of differential expression genes in the cortex of OCD patients. Among these risk genes of OCD, we found *KCTD12*, as a potential hub with kME = 0.81. Although none of the synapse organization genes fell under the OCD risk genes, one of them (TMEM108) had a central position in the network (kME = 0.91, degree = 60). The only differential expression gene in the cortex of OCD patients in the network was *FEZF2*, which is located at the border of the network (kME = 0.65, degree = 4). It is of note that *FEZF2* is a gene with a potentially high severity level in psychiatric disorders, as evaluated by our published database [38].

## 4. Discussion

In our current study, we studied the convergence and divergence of the genetic basis underlying AN and OCD from the gene and network levels. We have found that although AN and OCD patients exhibited similar cerebral transcriptomic alterations, their risk genes affected networks with distinct functions and spatiotemporal expression patterns. These results extend the understanding of the shared pathology [13] between the two diseases and partially explain why their symptoms resemble each other but are not identical [8,10].

In the above results, one of the most significant discoveries of this study is the inconsistency between the genome and transcriptome abnormality of AN and OCD: they are caused by distinct risk gene networks, but exhibit a similar transcriptome alteration. The shared transcriptomic patterns across psychiatric diseases have been demonstrated by the meta-analysis of Gandal et al. [39], who found that the prefrontal cortex of patients with autism, schizophrenia, bipolar disorder, and depression exhibited similar differential expression patterns, and their differential expression genes were enriched in similar coexpression modules. Although the expression data of AN and OCD [28] had a relatively smaller sample size, we found a convergent transcriptomic alteration between the two diseases by using the same approach (correlation analysis between logFC) as Gandal et al. This phenomenon approves of the current effort of cross-disorder studies that aim at common symptoms and pathologies shared by psychiatric disorders [40]. However, the divergent roles of risk genes in the different disorders seem contradictory to the convergent transcriptomic patterns. An integrative analysis by Shohat et al. [41] showed that rare mutations found in intellectual disability (ID), autism, and schizophrenia (SCZ) had distinct functions in chromatin organization and neuron development and exhibited different regions and cell-type enrichment. We also found that the AN and OCD risk genes were enriched in distinct coexpression modules. 

This contradiction may arise from various influencing factors. First of all, most of the psychiatric transcriptome analyses, including Jaffe et al. and Gandal et al., focused on the same region (prefrontal cortex), whereas genetic factors underlying different diseases pointed toward different brain regions [36,41,42,43]. Similarly, various imaging [44,45] and functional studies [46] also support the idea that the critical region and neural circuits of different diseases are distinct. It could be inferred that genetic variations first introduced transcriptomic abnormalities in the primary brain regions responsible for different diseases, and then caused additional influences in the prefrontal cortex, which are similar across diseases. Secondly, as pointed out by Gandal et al. [39], the linkage between genetic risk and expression alteration is complex and indirect, often a multi-step cascade of different biological processes. Among this cascade, a lot of influencing factors, both biological and environmental, are shared among psychiatric diseases. For example, chromatin modification has been indicated in the pathology of SCZ [47], autism [48], ID [49], OCD [50], and AN [51], and has the capacity of regulating gene expression levels [52]. Stress events and inflammation, two environmental factors that significantly impact the gene expression of central nervous system [53,54], contribute to the risk of various psychiatric diseases [55,56], especially AN [57] and OCD [58]. Lastly, damaging genetic variations do not necessarily lead to expression abnormality. As revealed by the probability of the loss-of-function intolerant (pLi) score [59] and haploinsufficiency score [60], a gene can be sensitive either to truncating variants or dosage change (i.e., expression alteration), or both, without apparent causative relationships. Thus, not all dosage changes of genes are pathologically meaningful, nor do all damaging genetic variations come into effect by causing dosage changes, which explains the divergence between risk genes and transcriptomic alterations.

Using integrative approaches, we found that AN and OCD had different coexpression patterns concerning developmental periods, brain regions, and cell types. Epidemiological studies have demonstrated that the onset age of AN was usually in late adolescence (16~18 years old) [61,62], consistent with our finding that AN risk genes were enriched in modules that peaked in adolescence. For OCD, its risk genes peaked in mid-prenatal, which might partially explain the existence of the early-onset subtype of OCD [63]. Although previous studies did not observe the differences in brain regions (both affecting cortical-limbic circuits [64,65]) or cell types (both affecting serotonergic neuron [66,67]) involved in AN and OCD, we showed that the risk genes of the two diseases have such a distinction. In fact, the AN risk genes mostly affect the subcortical regions and endothelium, whereas the OCD risk genes mainly affect cortices and neurons. These results suggest that the two diseases may have different “primary” regions and cells where the original dysfunctions emerge.

The more encouraging results came from the followed functional enrichment analysis and network analysis. By integration of developmental coexpression patterns, disease GWAS implication and disease transcriptomic alteration, we found that the AN risk genes enriched in the network of extracellular matrix (ECM) organization centered at *LAMB2* and *HTR1A*, whereas the OCD risk genes were enriched in that of synapse functions. Researchers have devoted themselves to studying the dysfunction of neurotransmitter systems for both AN and OCD [68], with a primary focus on the neuronal and synaptic functions [69]; however, a study on the impact of ECM and non-neuronal cell types is still lacking. These studies have been proven as fruitful and provided profound insights into the mechanism of the two diseases. However, our results suggest that applying this identical strategy on two different diseases may introduce some kinds of bias since the risk genes of AN show significant impacts on ECM organization as well as on non-neuronal cell types, such as the endothelium and astrocytes, which is distinct from that of OCD. Large amounts of evidence [70] show that ECM modulates various functions, such as stem cell differentiation, neuron migration, axonal tract formation, myelination, and synapse maturation, all of which serve as essential processes of neurodevelopment. Similarly, non-neuronal cell types modulate neurotransmitter and synaptic functions via molecule clearance, neurotrophic release, and other processes [71]. Both our study and previous studies [72,73] suggest that such critical processes have an essential role in the pathology of AN and should not be neglected. We also infer that such a divergence with OCD may be one of the essential mechanisms underlying the phenotypic difference of the two diseases.

We also notice that the current study has limitations in the inclusion of risk genes. Various genetic factors other than SNP, including de novo mutation and copy number variation, have a fundamental contribution to psychiatric diseases, yet they are excluded in the current study. This is partly because limited trio-based sequencing studies are available publicly for AN or OCD [50,74]. The GWAS used for the current study did not excluded co-morbidity, which might introduce bias into the identified risk genes. Furthermore, the transcriptome data of ED came from various subtypes of ED, with only three samples coming from classic AN patients. Thus, the result of the ED cortex expression might contain signals shared by different types of ED, instead of being specific to AN.

## 5. Conclusions

The AN risk genes were enriched in coexpression modules that were involved in extracellular matrix functions and highly expressed in the postnatal brain, limbic system, and non-neuronal cell types, whereas the OCD risk genes were enriched in modules of synapse function, the prenatal brain, cortex layers, and neurons. However, they led to similar prefrontal cortex expression alterations, which influenced synapse transmission. We have found that the functional and spatiotemporal enrichment patterns the AN and OCD risk genes were not identical, but they brought about similar expression alterations, which may be one of the reasons why they had similar but not identical clinical phenotypes.

## Data Availability

All data analyzed in this study are curated from public domain.

## Supplementary Materials

The following are available online at https://www.mdpi.com/article/10.3390/genes12040491/s1, Figure S1 Modules of AN and OCD had different preference on developmental periods A&B: GO-BP analysis for prenatal AN module and postnatal OCD module. C&D: Period-specific expression of AN and OCD module genes. Table S1 All AN and OCD risk genes included in the current study. Table S2 Full GSEA result.
