# Supplementary figures and images for: Dissection of the Genetic Association between Anorexia Nervosa and Obsessive–Compulsive Disorder at the Network and Cellular Levels
Source: Genes (Basel). 2021 Mar 27;12(4):491. doi: 10.3390/genes12040491 (PMC8065602; doi:10.3390/genes12040491)

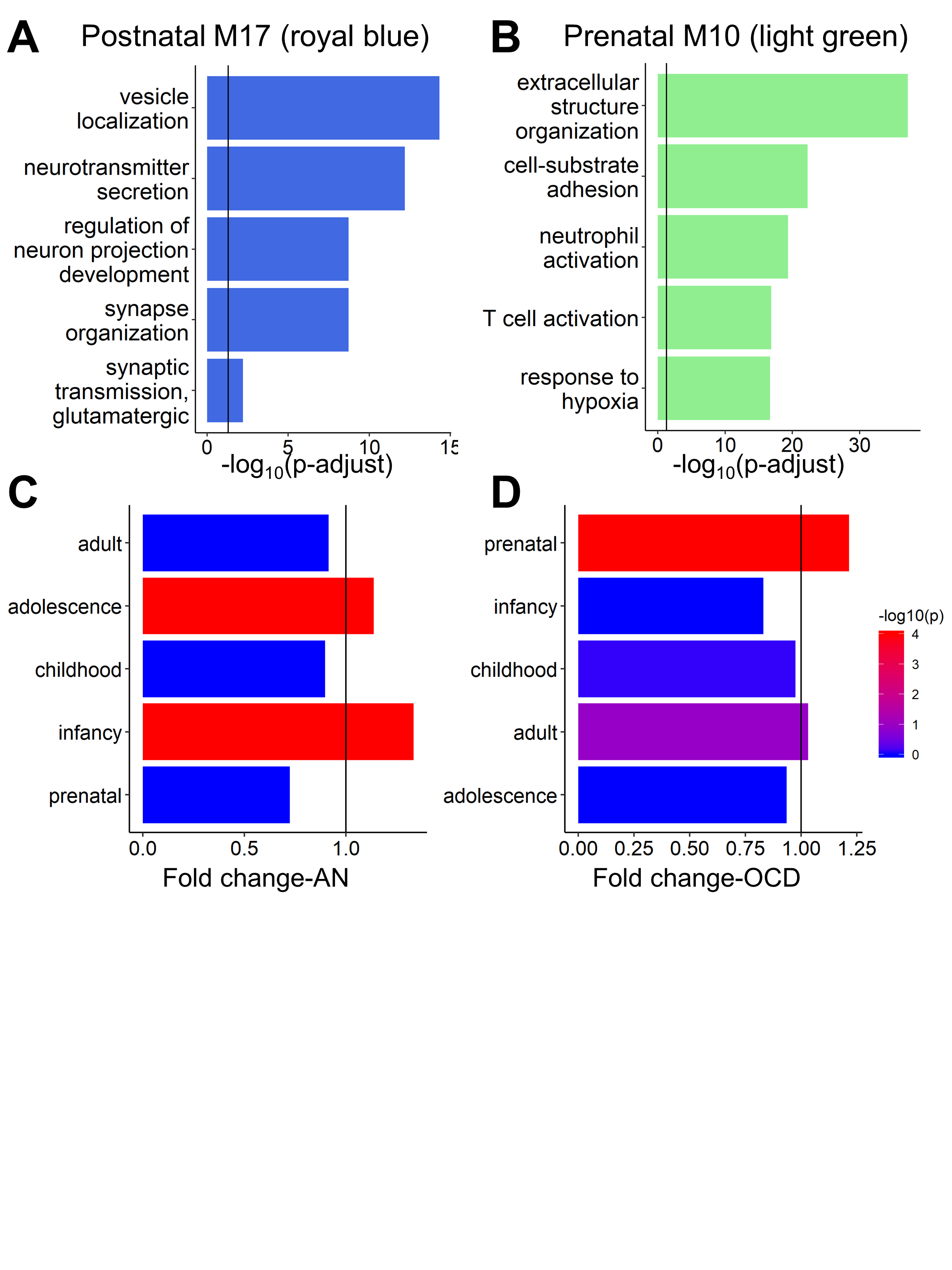

Supplement: Supplementary file 1 [file genes-12-00491-s001.zip › figures1.tif]
